#  Supplemented powdered coconut water (ACP-406^®^) promotes growth of goat secondary follicles and oocyte meiotic resumption

**DOI:** 10.21451/1984-3143-AR2019-0008

**Published:** 2019-11-18

**Authors:** Luana Mirela de Sales Pontes, Bruna Bortoloni Gouveia, Vanúzia Gonçalves Menezes, Vanessa Raquel Pinto de Barros, Ricássio de Sousa Barberino, Alane Pains Oliveira do Monte, Nathalie Jiatsa Donfack, Juliana Jales de Hollanda Celestino, Cristiane Clemente de Mello Salgueiro, José Ricardo de Figueiredo, Maria Helena Tavares de Matos

**Affiliations:** 1 Universidade Federal do Vale do São Francisco, Núcleo de Biotecnologia Aplicada ao Desenvolvimento de Folículos Ovarianos, Petrolina, PE, Brasil; 2 Universidade da Integração Internacional da Lusofonia Afro-Brasileira, Instituto de Ciências da Saúde, Acarape, CE, Brasil; 3 Universidade Estadual do Ceará, Rede Nordeste de Biotecnologia, ACP Biotecnologia, Setor de Inovação Biotecnológica, Fortaleza, CE, Brasil; 4 Universidade Estadual do Ceará, Faculdade de Medicina Veterinária, Laboratório de Manipulação de Oócitos e Folículos Pré-Antrais, Fortaleza, CE, Brasil

**Keywords:** caprine, ovary, *In vitro* culture, *In vitro* maturation

## Abstract

The objective of this study was to test the efficiency of powdered coconut water (ACP-406^®^) base-medium without or with the addition of supplements on *in vitro* culture of isolated goat secondary follicles. Follicles were cultured for 18 days in α-MEM or in ACP-406^®^, both without supplements (referred to as α-MEM and ACP, respectively), or both supplemented with BSA, insulin, transferrin, selenium, glutamine, hypoxanthine, and ascorbic acid (referred to as α-MEM^+^ and ACP^+^). Follicular morphology, antrum formation, follicular and oocyte diameter, levels of glutathione (GSH), and chromatin configuration after *in vitro* maturation were evaluated. At the end of culture, ACP-406^®^ base-medium (without or with supplements) showed a higher (P *<* 0.05) percentage of normal follicles than α-MEM (without or with supplements). Antrum formation was similar among α-MEM^+^, ACP and ACP^+^, and significantly higher than α-MEM without supplements. The follicular diameter was greater in ACP^+^ than α-MEM, and similar to other treatments. Moreover, fully and daily grown rates were higher (P *<* 0.05) in ACP-406^®^ base-medium (without or with supplements) than α-MEM (without or with supplements). Levels of GSH were similar between ACP^+^ and α-MEM^+^ treatments. Both ACP^+^ and α-MEM^+^ allowed meiotic resumption without a significant difference between the two groups. In conclusion, supplemented ACP-406^®^ base-medium maintained follicular survival and promoted the development as well as meiotic resumption of isolated goat secondary follicles cultured *in vitro* for 18 days.

## Introduction

*In vitro* culture of ovarian follicles has emerged as a potential reproductive technology to produce large numbers of mature oocytes that are capable of fertilization (Demeestere et al., 2005). Additionally, this contributes to a better understanding of the mechanisms involved in oocyte development and control of folliculogenesis (Silva et al., 2016). However, despite the considerable efforts aiming to improve this biotechnology, the success is still limited. One of the limitations could be the use of inappropriate culture medium. In this context, different types of culture media have been used to ensure the nutritional requirements of ovarian follicular growth and development in small ruminants such as alpha-minimum essential medium (α-MEM) (Cadenas et al., 2017; Menezes et al., 2017). However, the use of synthetic media could be expensive and less accessible. This has led researchers to investigate alternative and easily obtainable media such as powdered coconut water.

The coconut water solution has been successfully used for ovarian tissue transport, preserving preantral follicles at low temperature (4 °C) (Silva et al., 2000; Andrade et al., 2002; Lucci et al., 2004). Moreover, it has been used for *in vitro* culture of buffalo preantral follicles (Gupta et al., 2008) and bovine embryos (Blume et al., 1997a), as well as for *in vitro* maturation (IVM) of bovine oocytes (Blume et al., 1997b). However, besides coconuts are not universally available, the biochemical properties of its water can vary among fruits due the stage of maturity and environmental conditions (i.e. temperature, relative humidity, solar radiation, and soil chemical composition) in which the coconut-tree develops (Lima et al., 2010; Prades et al., 2012), which can directly affect its ability to support cells in *in vitro* systems. Additionally, the maintenance of the biological characteristics of coconut water during storage is restricted to a defined interval of time (Cardoso et al., 2005). Thereby, powdered coconut water (ACP^®^) has been developed. This product can be easily stored and transported, and after reconstitution, its biochemical characteristics are very similar to those found in fresh coconut water (Lima et al., 2010).

The ACP^®^ has been used for preserving sperm (caprine: Salgueiro et al., 2002; canine: Cardoso et al., 2007; capuchin monkey: Oliveira et al., 2010), and for short-term preservation of canine (Lima et al., 2010) and collared peccaries (Lima et al., 2014) ovarian tissue. Moreover, addition of 5% ACP^®^ to the IVM medium improved the rates of metaphase II oocytes in canine species (Silva et al., 2010). However, the effect of ACP^®^ base-medium on *in vitro* culture of goat secondary follicles is still unknown.

Apart from the base-medium, it is well known that *in vitro* culture of ovarian follicles could be affected by the presence or absence of different supplements such as insulin-transferrin-selenium (ITS), pyruvate, glutamine, hypoxanthine, and bovine serum albumin (BSA) (Silva et al., 2004). These authors have shown that the addition of the above substances in the culture medium improves the survival of goat preantral follicles enclosed in ovarian tissue compared to non-supplemented base-medium. Moreover, supplementation of the medium with BSA increases the antrum formation after culture of goat secondary follicles (Rodrigues et al., 2010).

Therefore, the aim of this study was to evaluate the *in vitro* effect of ACP^®^ (ACP-406^®^) base-medium without or with supplements (insulin, transferrin, selenium, glutamine, hypoxanthine, BSA, and ascorbic acid) on the morphology, growth, levels of reactive oxygen species, glutathione, and active mitochondria, and on the meiotic resumption of oocytes from isolated goat secondary follicles.

## Methods

Unless indicated, all chemicals used were purchased from Sigma Chemical Co. (St. Louis, MO, USA).

## Ovary collection

Ovaries (n = 80) were collected from 40 adults, cross-bred goats at a local slaughterhouse. Ovaries were washed once in 70% alcohol, and twice in HEPES-buffered minimum essential medium (MEM) supplemented with antibiotics (100 µg/mL penicillin and 100 µg/mL streptomycin). Thereafter, the ovaries were transported within 1 h to the laboratory in tubes containing MEM-HEPES with antibiotics at 4 °C (Chaves et al., 2008). The approval of the ethics committee was not required since the research involved tissues of slaughtered animals.

## Isolation and selection of caprine secondary follicles

Isolation, selection, culture and follicular evaluation were performed according to Cadenas et al. (2017). The surrounding fatty tissues and ligaments were stripped from the ovaries; large antral follicles and corpora lutea were removed. The ovarian cortical slices (1-2 mm thick) were placed in holding medium consisting of MEM-HEPES with antibiotics. Goat secondary follicles, approximately 250 μm, without antral cavity were visualized under a stereomicroscope (SMZ 645 Nikon, Tokyo, Japan) and mechanically isolated by microdissection using 26-gauge (26 G) needles. Follicles selected for culture showed an intact basement membrane, two or more layers of granulosa cells and a visible and healthy oocyte that was round and centrally located, without any dark cytoplasm.

## *In vitro* culture of caprine secondary follicles

After selection, the follicles were pooled and randomly divided into four groups (approximately 40 follicles/group): (1) α-MEM without supplements (α-MEM); (2) α-MEM supplemented with 3.0 mg/mL BSA + 10 ng/mL insulin + 2.5 μg/mL transferrin + 5 ng/mL selenium + 2 mM glutamine + 2 mM hypoxanthine + 50 ng/mL ascorbic acid (α-MEM^+^); (3) ACP-406^®^ (diluted in distilled water; pH: 7.40; osmolarity: 300 mOsm/L; ACP Biotecnologia, Fortaleza, Ceará, Brazil) without supplements (ACP); or 4) ACP-406^®^ supplemented with the same substances described for α-MEM^+^ (ACP^+^). The follicles were individually cultured at 39 °C under 5% CO_2_ in the air for 18 days in 100 μL droplets of culture medium under mineral oil in Petri dishes (60 × 15 mm, Corning, Sarstedt, Newton, NC, USA). Every two days, 60 μL of the culture media was replaced with fresh media.

## Morphological evaluation of follicle development

The morphology of all follicles was assessed every six days using a pre-calibrated ocular micrometer in a stereomicroscope (SMZ 645 Nikon) at ×100 magnification. Only those follicles showing an intact basement membrane with bright and homogeneous granulosa cells and an absence of morphological signs of atresia were classified as morphologically normal follicles. Follicular atresia was recognized when a darkening of the oocytes and surrounding cumulus cells or misshapen oocytes was noted. The rupture of the basement membrane was also observed and characterized as oocyte extrusion. The following characteristics were analyzed in the normal follicles: (i) antral cavity formation, defined as the emergence of a visible translucent cavity within the granulosa cell layers; (ii) diameter, measured from the basement membrane, which included two perpendicular measurements of each follicle; (iii) daily growth rate, calculated as the diameter variation during the culture period (18 days); and (iv) total growth rate, calculated as the diameter of normal follicles at day 18 minus the diameter of follicles at day 0. Moreover, after 18 days, all healthy follicles were mechanically opened with 26-G needles for oocyte recovery. The percentage of fully grown oocytes (oocyte ≥ 110 μm) was calculated as the number of acceptable quality oocytes (≥ 110 μm) recovered out of the total number of cultured follicles (×100).

## Assessment of intracellular levels of reactive oxygen species, glutathione and metabolically active mitochondria

After *in vitro* culture, the oocytes were recovered and intracellular levels of reactive oxygen species (ROS), glutathione (GSH) and mitochondrial activity were measured (Gouveia et al., 2016). Briefly, 2’,7’-diacetate-dichloro-dihydro-fluorescein (H2DCFDA; Invitrogen Corporation, Carlsbad, CA, USA), 4-chloromethyl-6,8-difluoro-7-hydroxycoumarin (Cell Tracker™ Blue; CMF2HC; Invitrogen Corporation), and Mitotracker Red Mitotracker™ Red, CMXRos, Molecular Probes, Melbourne, Victoria, Australia) were used to detect levels of ROS, GSH and mitochondrial activity with green, blue and red fluorescence, respectively. Approximately 10-15 oocytes per treatment were incubated in the dark for 30 min in PBS supplemented with 10 μM H2DCFDA + 10 μM of CellTracker Blue™ + 100 nM Mitotracker™ Red at 39 °C. After incubation, the oocytes were washed with PBS for 30 min and the fluorescence was observed under an epifluorescence microscope with UV filters (460 nm for ROS, 370 nm for GSH and 579 nm for active mitochondria). The fluorescence intensities (in pixel) of the oocytes were analyzed by Image J software (Version 1.41, National Institutes of Health).

## Maturation of caprine oocytes from *in vitro* cultured secondary follicles

*In vitro* maturation (IVM) was performed in the oocytes derived from *in vitro*-grown secondary follicles after 18 days of culture in the treatment that obtained the best results of follicular development to verify the ability of these oocytes to resume meiosis. For this, additional pairs of caprine ovaries (n = 20 ovaries) were collected and transported to the laboratory as described above. After 18 days of culture, all oocytes enclosed in healthy follicles were carefully collected with 26-G needles. Only oocytes ≥ 110 μm of diameter, with a homogeneous cytoplasm, and surrounded by at least 1 compact layer of cumulus cells were selected for IVM (Cadenas et al., 2017). Briefly, approximately 20 cumulus–oocyte complexes (COCs) per treatment were transferred to drops of 100 μL of maturation medium composed of tissue culture medium 199 (TCM 199) supplemented with 1 mg/mL BSA + 1 mM/mL pyruvate + 0.5 µg/mL recombinant follicle stimulating hormone (FSHr) + 5 µg/mL luteinizing hormone (LH) + 1 µg/mL 17β-estradiol + 10 ng/mL epidermal growth factor (EGF) + 50 ng/mL insulin growth factor-1 (IGF-1) + 100 µM/mL cysteamine under oil, and incubated for 32 hours at 39 °C under 5% CO_2_. After IVM, the oocytes were incubated in drops of PBS containing 10 mM Hoechst 33342 for 15 min at room temperature in the dark and visualized under fluorescence microscopy. The chromatin configuration was analyzed through observation of the intact germinal vesicle (GV), meiotic resumption [including germinal vesicle breakdown (GVBD) or metaphase I (MI)] or nuclear maturation (metaphase II - MII).

## Statistical analysis

Data from follicular survival, antrum formation, retrieval of fully grown oocytes and meiotic resumption after *in vitro* culture were expressed as percentage and compared by the chi-square test. Data from ROS, GSH, mitochondrial activity, follicular diameter, and growth rate were submitted to the Shapiro-Wilk test to verify normal distribution of residues and homogeneity of variances. Then, Kruskal-Wallis nonparametric test was used for comparisons. When main effects or interactions were significant, the means were compared by the Student-Newman-Keuls test. The results were expressed as the mean ± standard error of the mean (SEM) (P *<* 0.05).

## Results

### Follicular morphology and development after culture

**Figure 1:**
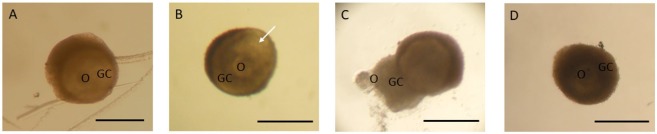
Morphologically normal secondary follicle at day 0 (A); antral (B), extruded (C) and atretic (D) follicles at day six of culture in ACP. GC = granulosa cell; O = oocyte; Arrow = antral cavity. Scale bar: 200 μm.

**Figure 2:**
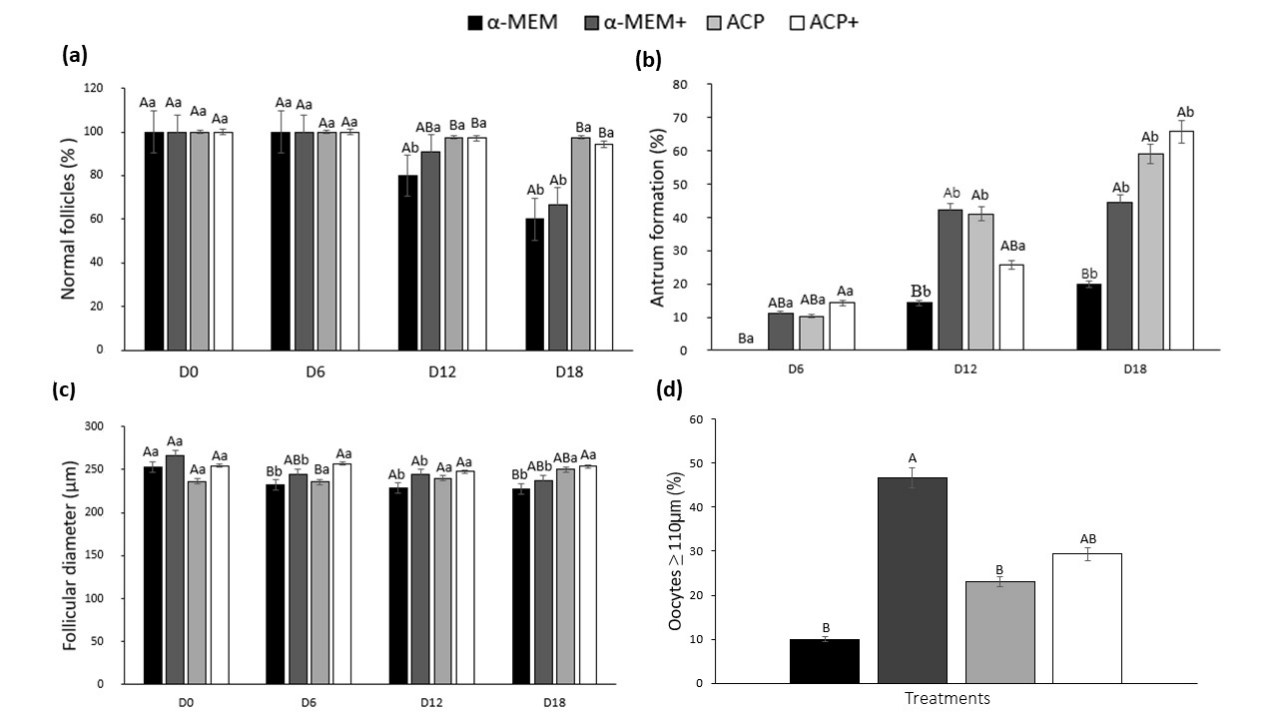
Percentage of morphologically normal follicles (a); antrum formation (b); follicular diameter (c), and fully grown oocytes after culture of secondary follicles (d). (^a,b^) Different letters denote significant differences among culture periods in the same treatment (P < 0.05). (^A,B^) Different letters denote significant differences among treatments in the same period (P *<* 0.05).

Morphologically normal secondary follicles showed centrally located oocytes and normal granulosa cells (Figure 1A). At day six of culture, antral (Figure 1B), extruded (Figure 1C), and atretic (Figure 1D) follicles could be observed. At the end of culture, the percentage of normal follicles was similar (P *>* 0.05) between ACP (97.4%) and ACP^+^ (94.3%), being both significantly higher than the other groups (60% for MEM and 66.7% for MEM^+^; Figure 2a). Moreover, only ACP (with or without supplements) treatments maintained the follicular survival throughout the culture period.

The rates of antral cavity formation increased significantly after 12 days in all treatments compared to day six except for ACP with supplement (Figure 2b). At the end of culture, the antrum formation was similar (P > 0.05) among α-MEM^+^ (44.4%), ACP (59%), and ACP^+^ (65.7%) and higher (P < 0.05) than α-MEM without supplements (20%).

At day 18, follicles cultured in ACP^+^ (253.9 µm) had a greater (P < 0.05) diameter than α-MEM without supplements (227.6 µm) and similar (P *>* 0.05) to other treatments (250 µm for ACP, and 237.3 µm for α-MEM^+^) (Figure 2c). The rates of total and daily follicular growth were similar between ACP and ACP^+^ treatments (P > 0.05), being both higher (P < 0.05) than α-MEM (with or without supplements) (data not shown). Additionally, at the end of culture, the percentage of fully grown oocytes (with diameter ≥ 110 µm) was similar (P > 0.05) between both supplemented treatments (α-MEM^+^ and ACP^+^) (Figure 2d). However, both α-MEM and ACP treatments showed a lower (P < 0.05) percentage of fully grown oocytes than α-MEM^+^ and similar (P > 0.05) to ACP^+^.

### Intracellular levels of ROS, GSH and metabolically active mitochondria

**Figure 3:**
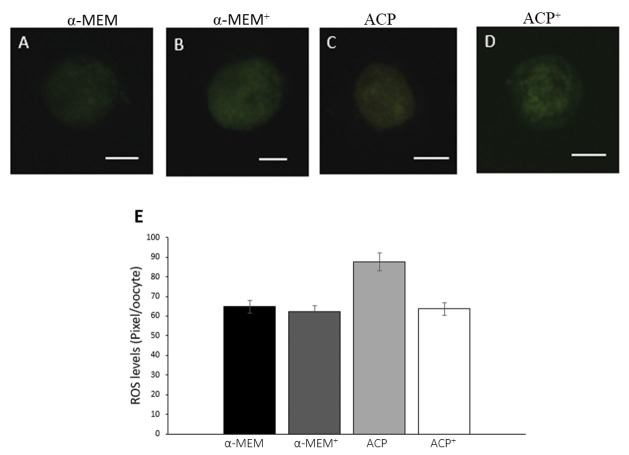
Detection of ROS in oocytes cultured in α-MEM without supplements (A); supplemented α-MEM (B); ACP without supplements (C) or supplemented ACP (D), and intracellular relative levels (pixel/oocyte) of ROS in oocytes after 18 days of culture in the different treatments (E). Scale bar: 50 μm.

**Figure 4:**
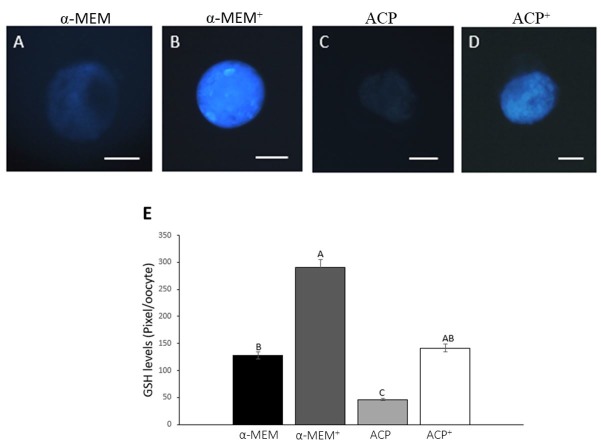
Detection of GSH in oocytes cultured in α-MEM without supplements (A); supplemented α-MEM (B); ACP without supplements (C) or supplemented ACP (D), and intracellular GSH relative levels (pixel/oocyte) in oocytes after 18 days of culture in the different treatments (E). Scale bar: 50 μm. (^A,B,C^) different letters denote significant differences among treatments (P *<* 0.05).

**Figure 5:**
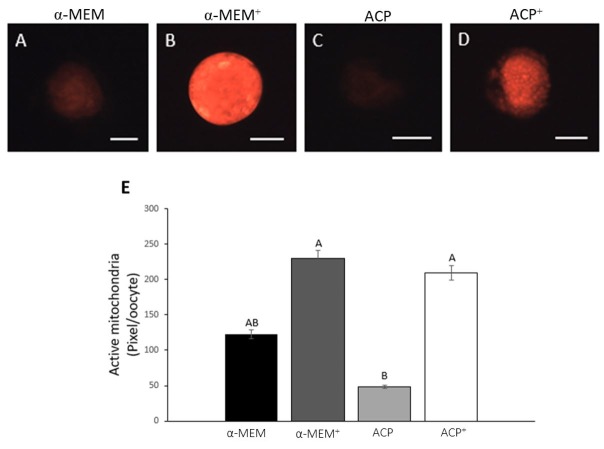
Detection of active mitochondria in oocytes cultured in α-MEM without supplements (A); supplemented α-MEM (B); ACP without supplements (C) or supplemented ACP (D), and intracellular mitochondrial activity relative levels (pixel/oocyte) in oocytes after 18 days of culture in the different treatments (E). Scale bar: 50 μm. (^A,B^) different letters denote significant differences among treatments (P *<* 0.05).

The levels of ROS were similar among all treatments (Figure 3). Oocytes from follicles cultured in both supplemented media (α-MEM^+^ and ACP^+^) had similar levels of GSH (P > 0.05) (Figure 4). Moreover, ACP without supplements presented the lowest (P < 0.05) level of GSH. All treatments showed a similar (P *>* 0.05) level of mitochondrial activity, except ACP without supplements, which was significantly lower than both supplemented media (α-MEM^+^ and ACP^+^; Figure 5).

### Chromatin configuration after IVM

**Figure 6:**
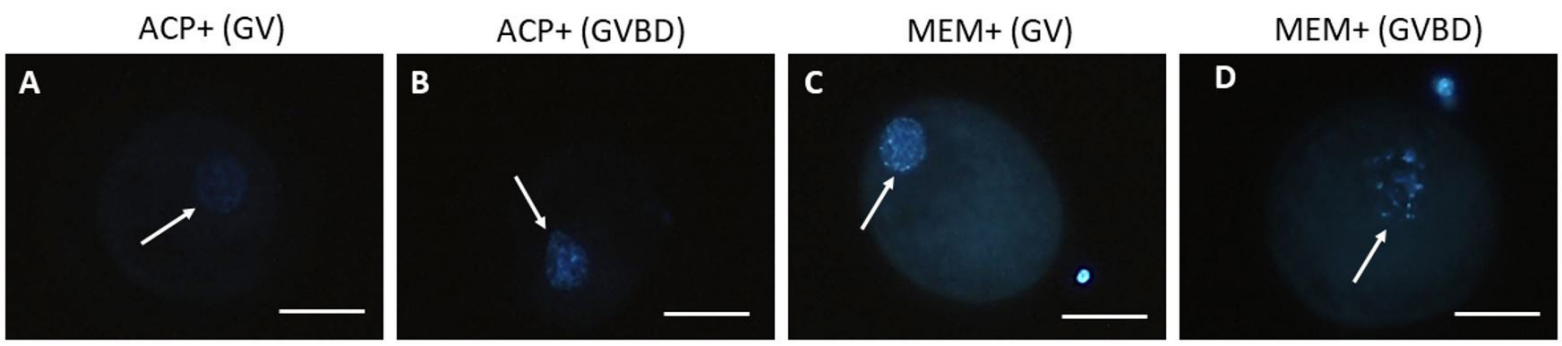
Epifluorescent photomicrographic images of caprine oocytes stained with Hoechst 33342 after IVM. Oocytes in GV (A, C) and GVBD (B, D) from follicles cultured in ACP or MEM with supplements (ACP^+^, MEM^+^). Arrow: nuclear chromatin. Scale bars: 50 μm (×100 magnification).

Oocyte meiotic competence was assessed by IVM of COCs obtained from secondary follicles that had been cultured in α-MEM^+^ or in ACP^+^. These treatments were chosen because they presented the highest rate of fully grown oocytes, and similar levels of GSH and active mitochondria. Both treatments showed a similar (P *>* 0.05) percentage of GV (α-MEM^+^: 60%; ACP^+^: 63.16%) and meiotic resumption rate (α-MEM^+^: 40%; ACP^+^: 36.84%) (Table 1; Figure 6).

**Table 1:** Meiotic status of goat oocytes derived from secondary follicles cultured in α-MEM^+^ or ACP^+^ medium.

**Treatments**	**GV**	**GVBD**	**MI**	**MII**
α-MEM^+^	60.0% (12/20)	25.0% (5/20)	10.0% (2/20)	5.0% (1/20)
ACP^+^	63.16% (12/19)	36.84% (7/19)	0.0% (0/19)	0.0% (0/19)

(P *>* 0.05). GV = germinal vesicle; GVBD = germinal vesicle breakdown; MI = metaphase I; MII = metaphase II.

## Discussion

This study investigated for the first time the effect of ACP-406^®^ base-medium on the *in vitro* development of goat secondary follicles followed by *in vitro* maturation of oocytes. Regardless of the supplementation, ACP-406^®^ base-medium showed the highest percentage of normal follicles compared to α-MEM after 18 days of culture. ACP-406^®^ is a rich nutrient medium composed of sugars (fructose, sucrose and glucose), amino acids (glutamic acid, arginine, leucine, valine, glycine, phenylalanine, serine), vitamins (folic acid, ascorbic acid, pantothenic acid and thiamine), lipids and electrolytes (sodium, calcium, iron, potassium). It is important to note that many substances are present in higher concentration in ACP-406^®^ than α-MEM such as folic acid, pantothenic acid and biotin. Some of the compounds present in ACP^®^ are well known as survival or antiapoptotic factors (ascorbic acid and folic acid), which could explain our results of follicular survival in ACP-406^®^ base-medium. It has been shown that folic acid decreased the amount of ROS in matured oocytes and increases the level of GSH thereby improving oocyte quality (Lazalde-Ramos et al., 2012). Moreover, ascorbic acid is one the most important oxygen scavenger in extracellular fluids, protecting the oocytes against oxidative stress (Wang et al., 2002). Some studies also showed that addition of ascorbic acid to the culture medium maintains follicular survival and promotes ovarian follicle growth (Sá et al., 2017; Tagler et al., 2014).

In this study, α-MEM without supplements showed the lowest antrum formation rate compared to the other treatments. Although α-MEM is a rich medium commonly used for *in vitro* culture of ovarian follicles, it requires additives beyond the standard medium nutrients to ensure follicle development including insulin, transferrin, selenium, glutamine, hypoxanthine, BSA, and ascorbic acid (Abedelahi et al., 2010; Rodrigues et al., 2010). Moreover, Rodrigues et al. (2010) observed an increase of caprine antrum formation and follicular diameter after the addition of BSA to the α-MEM medium. Therefore, the addition of supplements in the culture medium appears to be necessary to improve follicular development.

In our study, total and daily growth rates of the follicles were higher in ACP-406^®^ medium (with or without supplements) than α-MEM in the same condition. In buffalo, coconut water solution also increased follicular growth rates after *in vitro* culture (Gupta et al., 2008). Again, this result could be due to the nutrient composition of this medium such as sucrose and fructose. It has been shown that while glucose is the preferred energy substrate to support the gonadotrophin-induced differentiation of ovine granulosa cells *in vitro*, fructose and pyruvate represent alternative energy sources (Campbell et al., 2010).

Additionally, intracellular levels of GSH were similar between supplemented media (ACP^+^ and α-MEM^+^). The GSH may play an important role in cell protection against ROS during oxidative stress (Timme-Laragy et al., 2013). It has also been reported that ACP-109^®^ has an auxin in its composition called 3-indole-acetic acid (IAA) (Silva et al., 2011) and that IAA may act as an antioxidant by inhibiting peroxidation (Cano et al., 2003). Moreover, there are some antioxidant substances in ACP-406^®^ like ascorbic acid and folic acid (Leong and Shui, 2002) which could increase GSH production. We may suggest that the presence of IAA could increase GSH levels, which may improve the antioxidant defense and, therefore, support the follicle survival. Nevertheless, α-MEM also has antioxidant substances that may enhance the GSH levels (ascorbic acid and folic acid). In the current study, the levels of active mitochondria were also similar between ACP^+^ and α-MEM^+^. This result could be explained by GSH levels since the absence of GSH activity may lead to the alteration of cellular metabolism and consequently mitochondrial dysfunction (Fabbri et al., 2014). Our findings could also be related to the antioxidant (selenium, transferrin and ascorbic acid) or energetic (glutamine and BSA) potential provided by the supplements added to these culture media (Abedelahi et al., 2010; Rodrigues et al., 2010). Therefore, the high levels of GSH and mitochondrial activity of follicles cultured in both supplemented ACP and α-MEM could be considered as good markers of viability and energetic activity of mammalian oocytes (Castaneda et al., 2013).

Similar percentage of fully grown oocytes and meiotic resumption were observed between α-MEM^+^ and ACP^+^. These results indicate that ACP-406^®^ can promote oocyte development. Therefore, these findings are of a great importance because ACP-406^®^ base-medium could be an alternative to reduce the costs of the in *vitro* culture systems of ovarian follicles. However, more studies should be carried out to improve the *in vitro* maturation medium to obtain competent oocytes for subsequent *in vitro* fertilization and embryo production.

In conclusion, ACP-406^®^ (with or without supplements) maintained follicular survival and improved the development of goat secondary follicles after 18 days of *in vitro* culture. Moreover, supplemented ACP-406^®^ (ACP^+^) could be used as an alternative culture medium because it maintained the levels of GSH, ROS, metabolically active mitochondria and meiotic resumption like supplemented α-MEM (α-MEM^+^).

## List of abbreviations used

ACP: Powdered coconut water

ACP+: Supplemented powdered coconut water

ANOVA: Analysis of variance

BSA: Bovine serum albumin

COCs: Cumulus–oocyte complexes

EGF: Epidermal growth factor

FSHr: Recombinant follicle stimulating hormone

GSH: Glutathione

GV: Germinal vesicle

GVBD: Germinal vesicle breakdown

IAA: Indole Acetic Acid

IGF-1: Insulin growth factor-1

ITS: Insulin-transferrin-selenium

IVM: In vitro maturation

LH: Luteinizing hormone

MEM: Minimal Essential Medium

MI: Metaphase

MII: Metaphase II

PBS: Phosphate-buffered saline

ROS: Reactive oxygen species

SEM: Standard error of the mean

α-MEM^+^: Supplemented alpha minimal essential medium
